# What Is the Prognostic Value of the Pathologic Response after Neoadjuvant Radiotherapy in Soft Tissue Sarcoma? An Institutional Study Using the EORTC–STBSG Response Score

**DOI:** 10.3390/cancers16203449

**Published:** 2024-10-11

**Authors:** Anastasia Stergioula, Theodoros Kormas, Stefania Kokkali, Nikolaos Memos, Evaggelos Pantelis, Despina Pouloudi, Georgios Agrogiannis

**Affiliations:** 1 1st Department of Pathology, Medical School, National and Kapodistrian University of Athens, 157 72 Athens, Greece; dpouloudi@med.uoa.gr (D.P.); agrojohn@med.uoa.gr (G.A.); 2 Center of Radiotherapy, IASO General Hospital, 151 23 Athens, Greece; 3 Radiotherapy Department, Iatropolis Clinic, 115 21 Athens, Greece; vpantelis@med.uoa.gr; 4 Department of Orthopedic Surgery, Agios Savvas Anticancer Hospital, 115 22 Athens, Greece; thkormas@doctors.org.uk; 5 Oncology Unit, Department of Internal Medicine, Hippocratio General Hospital, National and Kapodistrian University of Athens, 157 72 Athens, Greece; stefkokka@med.uoa.gr; 6 2nd Department of Surgery, Medical School, Aretaieion Hospital, National and Kapodistrian University of Athens, 157 72 Athens, Greece; nikosmemos@aretaieio.uoa.gr; 7 Medical Physics Laboratory, Medical School, National and Kapodistrian University of Athens, 157 72 Athens, Greece

**Keywords:** soft tissue sarcoma, preoperative radiotherapy, neoadjuvant radiotherapy, pathologic response, EORTC response score

## Abstract

**Simple Summary:**

The pathologic response after neoadjuvant radiotherapy in soft tissue sarcoma of the extremities and trunk was evaluated using the EORTC-STBSG response score. The median percentages of viable cells, necrosis and fibrosis/hyalinization were 20%, 11% and 40%, respectively. A pathologic complete response, defined as ≤5% viable tumor cells, was achieved in 25% of cases. Local recurrence occurred in 33% of cases, with a significantly higher rate of 64% after R1 excision compared to 22% after R0 resection. Distant metastases were observed in 42% of patients, primarily in the lungs. The local recurrence free survival, distant metastasis free survival and overall survival rates were 65%, 54%, and 67% at 3-years, respectively. A correlation between tumor histological subtype, size and grade with outcome was observed. While the EORTC-STBSG response score did not correlate with clinical outcomes, resection specimens with ≤5% viable tumor cells were linked to improved outcomes.

**Abstract:**

**Background/Objectives:** The relationship between pathologic findings in soft tissue sarcoma (STS) after neoadjuvant treatment and oncological outcomes remains uncertain due to varying evaluation methods and cut-off values. This study aims to assess pathologic findings after neoadjuvant radiotherapy in STS using the EORTC-STBSG response score and evaluate its prognostic value. **Methods:** Clinical and outcome data from 44 patients were reviewed. Resected specimens were re-evaluated to measure viable cells, necrosis, fibrosis, and hyalinization. Local recurrence-free survival (LRFS), distant metastasis-free survival (DMFS), and overall survival (OS) were analyzed using Kaplan–Meier survival analysis. Cox proportional hazards regression was used for univariate and multivariate analyses to correlate outcomes with pathologic response. **Results:** The median percentages of viable cells, necrosis, and fibrosis/hyalinization were 20%, 11%, and 40%, respectively. A pathologic complete response (pCR), defined as ≤5% viable cells, was achieved in 25% of cases. Local recurrence occurred in 33% of cases, with a significantly higher rate of 64% after R1 resection compared to 22% after R0 resection. Distant metastases were observed in 42% of patients, primarily in the lungs. The 3-year rates for LRFS, DMFS, and OS were 65%, 54%, and 67%, respectively. A correlation between outcomes and tumor size, grade and histological subtype was observed. Classifying pathologic response by the EORTC-STBSG score failed to show an association with outcomes. Patients achieving pCR showed lower risk of LR and improved OS. **Conclusions:** While the EORTC-STBSG score did not show a prognostic value, resection specimens with ≤5% viable cells were linked to improved LRFS and OS.

## 1. Introduction

Soft tissue sarcoma (STS) comprises a heterogeneous group of rare tumors originating from mesenchymal cells [1,2,3]. Primary STS locations include the trunk, head and neck and abdomen, but the extremities are the most frequently affected sites. The management of localized STS typically involves a multimodal approach, incorporating surgery, radiotherapy, and chemotherapy (in the case of tumors with high risk of recurrence) [1,2,3]. Surgical resection with negative margins remains the cornerstone treatment of STS tumors. Radiotherapy (RT) is additionally used and can be administered in the neoadjuvant or adjuvant setting, offering similar outcomes but with different toxicity profiles [4,5]. While adjuvant RT offers the opportunity for a definitive histological assessment, the pathologic response to neoadjuvant RT can serve as a predictor of clinical outcomes. This is particularly evident in osteosarcoma patients receiving neoadjuvant chemotherapy, where a good pathologic response is associated with an 80% overall survival (OS) rate, compared to 50% in those who did not achieve such a response [6]. A 90% necrosis rate is the commonly accepted threshold for distinguishing good from poor responses [6]. The prognostic value of the pathologic response of STS tumors to neoadjuvant treatments remains unclear. Different factors have been used in the literature to determine the pathologic response, including the percentage of tumor necrosis [7,8,9,10,11,12,13,14,15,16,17], the number of viable cells [15,16,17,18,19,20], and the amount of fibrosis and hyalinization [15,17,20]. Moreover, no standard cut-off value has been established to assess the prognostic effect of pathologic response.

The European Organization for Research and Treatment of Cancer–Soft Tissue and Bone Sarcoma Group (EORTC–STBSG) has proposed a standardized approach for the histological examination of sarcomas, and a 5-tier score for assessing their pathologic response to neoadjuvant treatments [21]. The EORTC–STBSG response score is based on the percentage of stainable, potentially viable tumor cells, and differs from earlier methods that relied on tumor necrosis. Since tumor necrosis may be present in some STS tumors at diagnosis, evaluating the number of stainable cells in the resected specimen seems to offer a more reliable measure of the pathologic response to neoadjuvant treatments. However, while this response score was thoughtfully designed, it failed to demonstrate a prognostic value in three retrospective studies [15,16,17].

In this study, we aimed to determine the pathologic response of STS tumors located in the extremities and trunk that received neoadjuvant radiotherapy and definitive surgery at a single institution, and to evaluate the prognostic value of the EORTC–STBSG response score.

## 2. Materials and Methods

### 2.1. Patient Population

A retrospective review of our database was conducted, including 102 patients with histologically confirmed primary STS who underwent neoadjuvant RT followed by surgical resection between 2011 and 2023. Neoadjuvant RT was recommended after a multidisciplinary discussion that evaluated factors such as the risk of positive margins, tumor location, grade, size, and histopathologic subtype. Patients were excluded if they were under 18 years of age, had recurrent sarcoma, metastatic disease at diagnosis, retroperitoneal STS, bone sarcomas (e.g., Ewing’s sarcoma, chondrosarcoma, osteosarcoma), and benign or intermediate histological subtypes such as aggressive fibromatosis, desmoid tumor, or dermatofibrosarcoma protuberans. Patients who received neoadjuvant chemotherapy or other than the standard fractionation scheme of neoadjuvant RT were also excluded. From this cohort of 52 patients, complete resected specimen slides were available for re-evaluation for 44 patients who were included in the final analysis. It is noted that tumors were resected with a wide margin of surrounding healthy tissue, aiming to achieve complete resection with negative surgical margin. No specific recommendation for the width of that margin was used, in accordance with the current guidelines. Tumor margin status was reported using the Union for International Cancer Control (UICC) R + 1 mm classification, where R0 indicates a margin of more than 1 mm between the tumor and healthy tissue, R1 indicates a margin of less than 1 mm and R2 indicates macroscopically involved margins. Staging was conducted according to the 8th edition of the American Joint Committee on Cancer (AJCC) system. Histologic type and grade were reported using the World Health Organization (WHO) classification and the French National Federation of Cancer Centers (FNFCC) system, based on biopsy results obtained prior treatment. Molecular features were not used alongside with histopathological findings to aid the diagnosis of sarcoma histologic subtype. Grade 2 and 3 tumors in the FNCLCC system were classified as “high grade”, whereas grade 1 tumors were classified as “low grade”.

### 2.2. Radiotherapy Treatment Protocol

Radiotherapy was delivered using 6MV X-ray linear accelerator platforms, with all patients positioned supine using customized immobilization devices to optimize treatment setup reproducibility. Treatment planning was based on simulation Computed Tomography (CT) scans of the patient’s anatomy with 3 mm thick slices. Target delineation was performed following the established consensus guidelines [22,23]. In detail, the Gross Tumor Volume (GTV) was defined using contrast-enhanced T1-weighted (T1w) and T2-weighted (T2w) MRI images registered on the simulation CT. The Clinical Target Volume (CTV) was generated by expanding the GTV by 1–1.5 cm axially and 2–3 cm longitudinally, depending on tumor size and grade, while respecting anatomical barriers (e.g., bone and facia) and ensuring that the peritumoral edema identified on the T2w MRI images was encompassed. The Planning Target Volume (PTV) was then defined by isotropically expanding the CTV by 5 mm to account for residual positional uncertainties. A dose of 50 Gy in 25 fractions was delivered using either 3D conformal radiation therapy (3DCRT) or Volumetric Modulated Arc Therapy (VMAT) techniques.

### 2.3. Pathologic Evaluation

The surgical specimens were handled according to the guidelines of the College of American Pathologists [24]. Representative sections of each tumor were taken to include one section per centimeter of maximum dimension. This resulted in a variable number of sections, with an average of 12 sections per patient in the studied population. All specimens were re-analyzed by two experienced sarcoma pathologists who were blinded for clinical outcome. The percentage of viable tumor cells, necrosis, and fibrosis/hyalinization was assessed for each slide, and the overall percentage of each component was calculated for the entire specimen, so that the sum of the three components totaled 100%. Viable cells were identified as stainable, atypical tumor cells characterized by hyperchromatic nuclei, smudged and clumped chromatin, cellular and nuclear pleomorphism, large nucleoli, atypical and bizarre mitoses, loss of tissue polarity, and increased cellular size. Necrosis was quantified as confluent areas composed of necrotic tumor cells (preserved cellular outline “ghost” cells with loss of nuclear stain) amongst other degenerate cells, karyorrhectic nuclear debris, and polymorphonuclear neutrophil infiltrate. Fibrosis/hyalinization were quantified as hypocellular areas with deposition of dense, amorphous, uniform eosinophilic material, with/without dense collagenous matrix containing fibroblasts.

### 2.4. Pathologic Response Assessment

The pathologic response to neoadjuvant treatment was classified according to the EORTC–STBSG score, which categorizes the percentage of viable cells in the resection specimen as follows: Grade A: no viable tumor cells; Grade B: single viable tumor cells or small clusters (overall less than 1% of the specimen); Grade C: ≥1% to <10% viable tumor cells; Grade D: ≥10% to <50% stainable tumor cells; Grade E: ≥50% viable tumor cells [21]. In addition, for reasons of comparison with the literature, the pathologic response was assessed using the percentage of necrosis, as well as the amount of fibrosis/hyalinization found in the resected specimen. Pathologic complete response (pCR) was defined as less than or equal to 5% viable tumor cells. A combination of the percentage of viable tumor cells and treatment related changes (i.e., fibrosis/hyalinization) was also used for pathologic response evaluation, classifying the response as “very good” or “good” if at least 10% of the tumor mass exhibited treatment related changes, with “very good” indicating less than 10% viable tumor cells and “good” indicating 10% to 50% viable tumor cells. Patients who did not meet these criteria were classified as non-responders.

### 2.5. Study Objectives

The scope of the study was to assess the prognostic value of pathologic response in STS tumors after neoadjuvant RT. The evaluated oncologic outcome parameters included the local recurrence-free survival (LRFS), the distant metastasis-free survival (DMFS), and the overall survival (OS). LRFS was defined as the time from surgery to the occurrence of local recurrence (LR) during follow-up, either suspected based on imaging or confirmed by biopsy. DMFS was measured from the time of surgery to the development of distant metastasis (DM). OS was calculated from the time of surgery to either the last follow-up or death. Besides pathologic treatment response, association of the oncologic outcomes with the clinicopathological characteristics of the cohort such as patient age at diagnosis, tumor size (defined as tumor’s largest diameter measured at the diagnostic MRI scan), tumor grade, histologic subtype, resection margin, and neoadjuvant treatment protocol was studied.

### 2.6. Statistical Analysis

Categorical variables were reported as counts and percentages. Continuous variables were reported as median and inter quartile range (IQR) and compared using Kruskal–Wallis rank sum tests. Survival analysis was performed using the Kaplan–Meier method. Lost to follow-up patients were censored. Univariate and multivariate analyses were performed using Cox proportional hazard models to identify factors that influence the studied oncological outcome parameters. Hierarchical regression was used to determine the most relevant independent variables included in the statistical analysis. The proportional hazard assumption was evaluated using the scaled Schoenfeld residuals. Among the studied parameters, patient age, the percentage of tumor necrosis and the amount of fibrosis/hyalinization were considered as continuing variables, whereas tumor size was stratified in two groups using a threshold of 10 cm. The percentage of viable tumor cells was classified in five groups using the EORTC–STBG response scale. A *p*-value of less than 0.05 (two sided) was considered significant, whereas a *p*-value of less than 0.1 (two sided) was considered near significant. Statistical analyses were performed using RStudio version 2024.04.2 (Posit software, PBC, Boston, MA, USA).

## 3. Results

### 3.1. Patient Population and Tumor Characteristics

The demographic and clinicopathological details of the studied patient cohort are presented in Table 1. The median age of the patients was 68 years (IQR: 56–74 years), with 20 females and 24 males. In 38 patients (86%) the STS was located on the lower limb, in five patients (11%) on the upper limb, and in one patient, it was located on the trunk. Tumor size was less than or equal to 10 cm in 23 patients (52%) and greater than 10 cm in 21 patients (48%). The STS histological subtype was undifferentiated pleomorphic sarcoma (UPS) in 23 patients (52%), myxoid liposarcoma (MLS) in seven patients (16%) and myxofibrosarcoma (MFS) in six patients (14%). Other histological subtypes less frequently found were liposarcoma, dedifferentiated liposarcoma and malignant peripheral nerve sheath tumor. Thirty-six patients (82%) had high-grade tumors, while eight patients (18%) had low-grade tumors. RT was delivered using standard 3DCRT technique in 27 patients (61%), while the remaining 17 patients received VMAT. Definitive surgery was performed at a median of 60 days (IQR: 40–71 days) after RT completion by dedicated sarcoma surgeons. R0 resection was achieved in 33 patients (75%), whereas R1 in 11 patients (25%). None of the analyzed patients had had an R2 resection.

### 3.2. Post Treatment Pathological Characteristics

Table 2 presents the measured pathologic characteristics of the resected tumors stratified by histological subtype. Overall, the median percentage of viable cells was 20% (IQR: 6–51%), of necrosis 11% (IQR: 2–40%) and of fibrosis/hyalinization 40% (IQR: 22–84%). Pathological findings were found to depend on the histological STS subtype. The MFS tumors were found to have the largest proportion of viable tumor cells, presenting a median value of 64% (IQR: 43–80%), compared to 20% (IQR: 10–45%) measured for the UPS and 10% (IQR: 7–12%) for the MLS tumors (*p* = 0.069). The median percentage of necrosis was 32% (IQR: 9–49%) for the UPS, which is significantly higher (*p* = 0.041) compared to 5% (IQR: 3–6%) and 1% (0–7%) for the MLS and MFS tumors, respectively. The specimens from the MLS tumors were found to have increased fibrosis presenting a median value of 85% (IQR: 81–87%), which is significantly higher (*p* = 0.044) compared to 35% (IQR: 21–49%) found in the specimens from UPS tumors and 21% (IQR: 12–36%) found in the specimens from MFS tumors.

**Figure 1:**
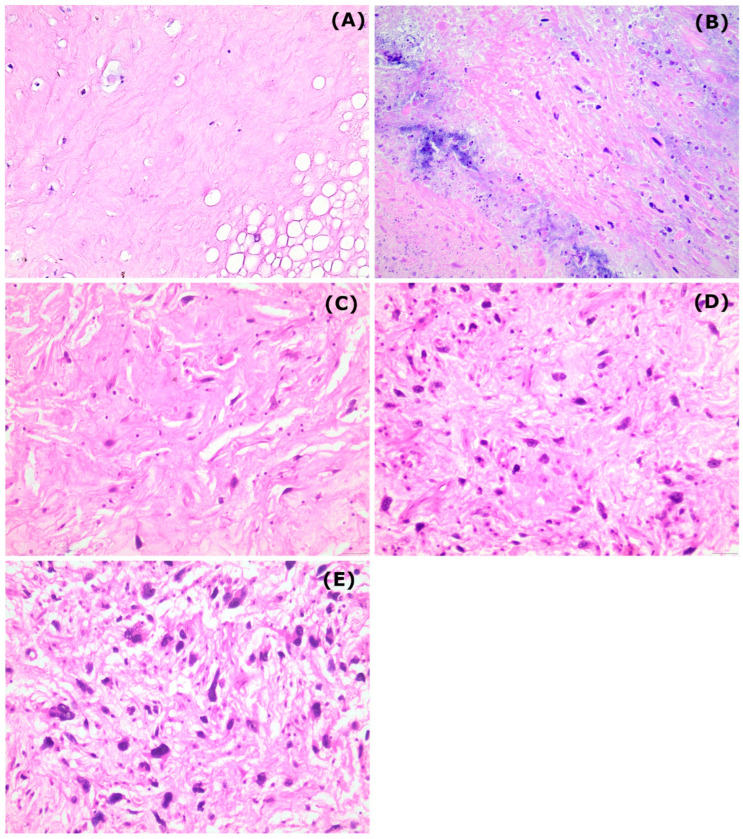
Representative figures from the different EORTC–STBSG grades corresponding to the outcome of radiotherapy (eosin–hematoxylin stain, 200× original magnification). (**A**) collagenous stroma with complete absence of neoplastic cells, (**B**) only scattered, single viable cells into a necrotic and collagenous stroma, (**C**) tumor cells interspersed throughout the stroma, occupying less than 10% of the area, (**D**) tumor cells can be easily identified, with a diffuse growth pattern occupying less than 50% of the area and (**E**) poor response to radiotherapy showing increased population of viable tumor cells occupying more than 50% of the area.

The pathologic treatment response classified according to the EORTC–STBSG score, stratified by histological subtype is also presented in Table 2. Most cases were classified as Grade D (41%), followed by Grade E (30%). Complete eradication of tumor cells (Grade A) was observed in only 7% of the cases. Pathologic complete response was observed in 11 patients (25%). A “very good” pathologic response was obtained in 13 patients (30%), a “good” response in 17 (39%), whereas 14 patients were classified as non-responders. In Figure 1 example cases categorized as grade A–E according to the EORTC–STBSG score are presented.

### 3.3. Follow-Up and Clinical Outcome

Out of 44 patients, one was lost on follow-up resulting in a cohort of 43 patients used for oncologic outcome evaluation. The median follow-up since surgery was 26 months (IQR: 9–58 months). At the last follow-up, 17 patients (40%) had no evidence of disease. Fourteen patients (33%) developed local recurrence. The median local recurrence free interval was 14 months (IQR: 6–51 months). Distant metastases occurred in 18 patients (42%). Of these, 15 patients (83%) had metastases in the lungs, one patient (6%) in both the lungs and brain, and two patients (11%) had metastases in the bones. The median metastasis-free time interval was 12 months (IQR: 5–51 months).

Tumor size was found to significantly affect the risk of DM and OS, but not resection margin and LR risk. Patients presenting with tumors larger than 10 cm were found to exhibit a 60% risk of developing DM compared to 26% for those with tumors ≤ 10 cm (*p* = 0.025). Additionally, patients with tumors smaller than 10 cm showed improved OS compared to those with larger tumors (*p* = 0.008). Out of the 36 patients with high-grade tumors, 13 relapsed locally (37%), and 17 developed DM (49%). In contrast, among the eight patients with low-grade tumors, 1 had LR (13%) and 1 had distant progression. On the last follow-up, 13 patients (37%) died of the disease, all of whom had high-grade tumors. Resection margin significantly affected the risk of LR, with seven out of 11 patients (64%) who had an R1 margin had a LR, compared to seven out of 33 patients (22%) with an R0 margin (*p* = 0.022).

**Figure 2:**
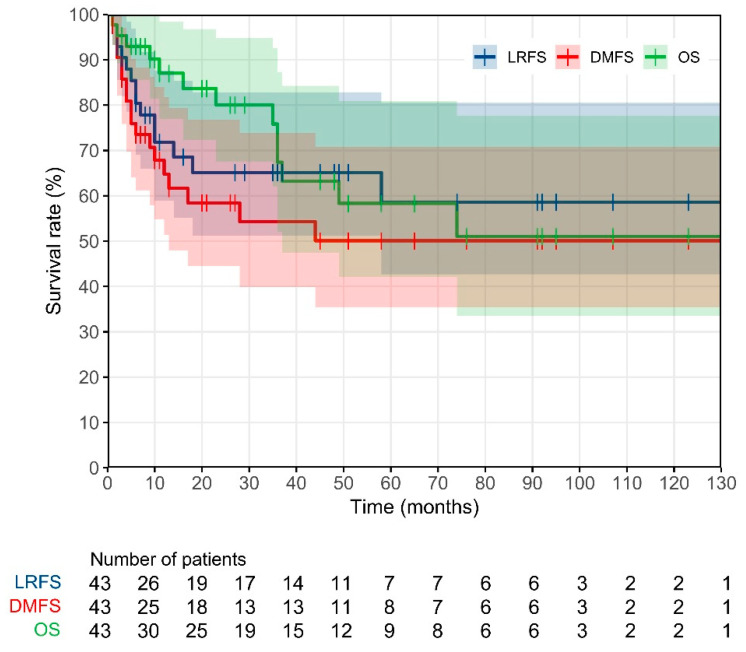
Kaplan–Meier curves for local recurrence free survival (LRFS), distance metastasis free survival (DMFS) and overall survival (OS) of the studied patient population.

Figure 2 shows the Kaplan–Meier curves for LRFS, DMFS and OS. As can be seen, the 3-year LRFS, DMFS and OS rates were found to be equal to 65%, 54% and 67%, respectively.

### 3.4. Prognostic Value of Clinicopathological Characteristics and Pathologic Response

Univariate and multivariate analyses were performed using Cox proportional hazard models to evaluate the correlation of the clinical tumor characteristics and the pathologic response with the LRFS, DMFS and OS outcomes of the patient cohort studied. Table 3 presents the results of the multivariate analysis, including only the variables that were found to be either significant or nearly significant. Univariate analysis identified patient age and resection margin as the only statistically significant predictors of local tumor recurrence, with increased age and an R1 margin associated with a higher recurrence risk (*p* = 0.006 and *p* = 0.040, respectively). Multivariate analysis confirmed the significance of patient age on LR and identified tumor grade as a contributing factor, with higher grades associated with an increased LR risk. While an R1 margin was associated with higher risk of LR it was of reduced significance in multivariate analysis (*p* = 0.322). The risk of DM was found to be associated with the tumor size and tumor grade, in both univariate and multivariate analyses. Tumors larger than 10 cm and high-grade tumors were found to be associated with an increased risk of developing distant metastasis (Table 3). Patient age and tumor size were identified as unfavorable factors for OS in both univariate and multivariate analyses. Additionally, histological subtype also influenced OS, with MFS exhibiting a higher hazard compared to UPS, although this finding was of near significance (*p* = 0.096).

Classifying pathologic response according to the EORTC–STBSG score did not show a significant correlation with oncological outcomes in either univariate or multivariate analyses. The percentage of tumor necrosis was associated with OS but not with local or distant disease progression (Table 3). The percentage of fibrosis/hyalinization in the resected tumor was not correlated with oncological outcomes in univariate and multivariate analyses. Although achieving a pCR was linked to a lower risk of LR and improved OS in multivariate analysis, these findings did not reach statistical significance (*p* = 0.195 and *p* = 0.183, respectively). Finally, categorizing patients as very good, good, or non-responders based on pathologic findings did not demonstrate prognostic value for oncological outcomes.

## 4. Discussion

Identifying early predictors of clinical outcomes following neoadjuvant RT for STS would be advantageous. If reliable, these predictors could serve as early primary endpoints in neoadjuvant trials and help identify patient subgroups that may benefit from treatment intensification. The EORTC–STBSG has proposed a standardized approach for assessing and reporting STS resection specimens after neoadjuvant therapy, including a 5-tier response score based on viable tumor cells. In this study, we evaluated the pathologic response of STS tumors after neoadjuvant RT using the EORTC–STBSG score and examined its correlation with the oncological outcome.

In the analyzed cohort, the overall median percentage values of viable cells, necrosis and fibrosis including hyalinization were 20%, 11% and 40%, respectively. The pathologic response was found to depend on histologic subtype. Forty-one percent of the cases were classified as Grade D in the EORTC–STBSG response score followed by Grade E (30%). Complete eradication of tumor cells (Grade A) was observed in only 7% of the cases. Local recurrence occurred in 33% of cases, with a significantly higher rate of 64% after R1 resection compared to 22% after R0 resection. Distant metastases were observed in 42% of patients, primarily in the lungs. The 3-year rates were 65% for LRFS, 54% for DMFS, and 67% for OS. It is noted that while DMFS and OS are in agreement with the corresponding survival data reported in the literature, the presented LRFS rate at 3 years falls on the lower end compared with the published data. Nevertheless, the presented LRFS, DMFS and OS align well with the published data at the 5-year follow-up [5,12,25].

Table 4 compares the pathologic findings of this study with previous published results, stratified by histologic subtype. An overall good agreement between the data in this study and the literature can be observed, further confirming that pathologic response varies depending on the histological subtype [17,25,26]. UPS tumors show a partial response to neoadjuvant treatment, with more than 10% viable tumor cells in the evaluated specimens, along with relatively increased necrosis and fibrosis/hyalinization. MFS tumors exhibited a higher number of viable tumor cells, a low percentage of necrosis, and relatively increased fibrosis/hyalinization. On the contrary, MLS tumors responded to neoadjuvant RT showing a small number of viable cells and extensive fibrosis/hyalinization, while necrosis is generally not observed. These findings are consistent with the growing body of literature highlighting the unique radiosensitivity of MLS, as evidenced in both radiological and pathologic responses to neoadjuvant treatments [25,27,28]. The mechanisms contributing to this radiosensitivity include vascular damage, adipocyte maturation, and a decrease in myxoid stroma [29,30].

Pathologic findings may depend on the timing of surgery following RT. Surgical resection of sarcoma tumors is typically performed 4 to 8 weeks after neoadjuvant RT. In this study, surgical resection was performed, on average, 8 weeks post RT, which is on the large delay side. However, the pathological findings are consistent with the corresponding data reported in the literature (see Table 4). The timing of surgical resection following neoadjuvant RT has not been found to impact clinical outcomes. In a study by Louie et al. [31], patients were stratified based on the time interval between RT and surgery (<6, 6–8, and >10 weeks), and no significant differences in recurrence or survival rates were observed. Additionally, an analysis of the National Cancer Database concluded that “clinicians may delay surgery up to 120 days after radiation to reduce the risks of wound complications and address modifiable comorbidities without affecting overall survival” [32].

Given the prognostic value of the pathologic response on the oncologic outcome, the data presented in Table 4 may be used to tailor preoperative treatment protocols based on histological subtype [25,28]. Indeed, the DOREMY trial recently reported the successful de-escalation of the preoperative radiotherapy dose for MLS tumors from 50 Gy to 36 Gy, based on their excellent pathologic and radiographic responses [33]. In contrast, for UPS and MFS tumors, which showed increased viable cells in the resected specimen, enhanced radiotherapy effectiveness using simultaneous integrated boost RT techniques [34], dose hypo-fractionated protocols based on the lower a/b ratio of STS tumors [35,36], or a combination with nanoparticle radiosensitizers may be warranted [37,38].

Local control in STS remains crucial, as recurrences have been linked to poor survival outcomes [13]. In our study, the impact of surgical margins aligned with these earlier findings. While the univariate analysis identified resection margin as statistically significant predictor of LRFS, this study was underpowered to detect a LR association with margin status in multivariate analysis. Higher-grade tumors were linked to an increased risk of local recurrence and distant metastasis. Additionally, a correlation was observed between histologic subtype and overall survival. Tumor size was found to affect both DMFS and OS, with larger tumors associated with a higher risk of distant metastasis and poorer overall survival, though it did not significantly impact LRFS. These findings are in agreement with results published in the literature [10,12,15,16,17,19,39]. It is noted that tumor size was evaluated in this study at diagnosis in accordance with standard clinical practice. Therefore, treatment related changes in tumor size (e.g., tumor shrinkage typically observed in MLS tumors during radiotherapy [30]) were not included in our analysis. Other studies have investigated the correlation of clinical outcome with tumor size evaluated post radiotherapy and just before surgery [16,27]. Reijers et al. [16] found that radiological response was significantly correlated with a lower percentage of viable cells and necrosis, but a higher percentage of fibrosis. However, no correlation of radiologic response with oncologic outcomes was observed. Incorporating changes in tumor size from preoperative RT into a prognostic system is challenging, as factors such as cystic transformation, hemorrhage, and necrosis can lead to an increase in size, instead of shrinkage, although the tumor is responding.

The EORTC–STBSG defines pathologic response to neoadjuvant treatment using a 5-tier score based on the percentage of viable tumor cells. Classifying the pathologic response of our patient cohort using this score did not reveal any prognostic value regarding oncologic outcomes. Two retrospective studies by Schaeffer et al. [15] and Reijers et al. [16] involving 100 and 107 STS patients of the extremities and trunk treated with neoadjuvant RT, respectively, also found no correlation between the EORTC–STBSG score and oncologic outcomes. Similarly, Boxberg et al. [17], in a study group of 64 patients, including 30 patients of a prospective monocentric clinical phase II study, with extremital and truncal STS treated with neoadjuvant RT monotherapy found no association between this scoring system and oncologic outcomes. Furthermore, another study that used the EORTC–STBSG score to assess pathologic response in extremity STS patients treated with neoadjuvant hyperthermic isolated limb perfusion followed by delayed surgical resection also failed to show any correlation with LRFS or OS [40]. While it seems intuitive that a Grade A pathologic response (complete eradication of viable tumor cells in the resected specimen) would be associated with improved oncologic outcomes, all studies have failed to demonstrate a correlation between the EORTC–STBSG score and oncologic outcomes. This lack of correlation could be attributed to the small number of patients achieving a Grade A response. In our study, only 7% of patients had a Grade A response, which aligns with the corresponding values reported in the literature, ranging from 0% to 9% [15,16,17]. As a result, these studies may be underpowered to detect the potentially improved outcomes in patients with complete tumor cell eradication in resected specimens.

The criteria for defining pathologic response after preoperative treatment have been widely debated. In this study, we defined pCR as having ≤5% viable tumor cells. A pCR was achieved in 25% of the analyzed patient cohort, consistent with the corresponding findings reported in the literature [15,16,17,19]. While achieving pCR was associated with improved LRFS and OS, this correlation did not reach statistical significance, likely due to the small cohort size. Moreover, patients achieving a pCR had 21% more R0 resections compared with patients who did not. Bonvalot et al. [19] conducted a large retrospective study involving 330 patients treated with neoadjuvant chemoradiotherapy (CRT) (*n* = 110) or RT (*n* = 222). The authors defined pCR as having ≤5% viable cells or ≥95% necrosis/fibrosis and found that patients with pCR had improved local relapse, distant metastasis, disease free survival and overall survival rates at 3 years [19].

The impact of necrosis after preoperative treatment has yielded conflicting results. Salah et al. [8], conducted a large systematic review and meta-analysis of 21 studies involving 1663 mostly high-grade extremity STS patients. The authors revealed that <90% necrosis after neoadjuvant therapy (including both RT and CRT) is a significant predictor of decreased disease-free survival and OS [8]. However, other studies have shown that increased necrosis is an unfavorable parameter for OS. Reijers et al. [16] and Schaefer et al. [15] evaluated the pathologic response in 107 and 100 STS patients receiving preoperative RT, respectively, and found that higher levels of necrosis correlated with reduced OS. Gannon et al. [12], evaluated the pathologic response of 167 STS patients and found that increased necrosis was correlated with worse LRFS, DMFS and OS. Our study supports these findings, further confirming that increased necrosis is linked to decreased OS. This could be attributed to the fact that necrosis is primarily an inherent adverse tumor characteristic; a portion of the necrosis in STS is pre-existing and plays a crucial role in determining tumor grade, with higher grades linked to worse outcomes [12,26]. To date, it remains difficult to distinguish pre-existing necrosis from treatment-induced necrosis. Moreover, necrosis is a definition of a moment, which challenges its measurement accuracy and robustness. In this work no comparison was made between treatment-induced necrosis and initial necrosis observed in core biopsies at diagnosis, as necrosis on core biopsies is considered an inaccurate estimation of the necrosis of the whole tumor. Mullen et al. [7] appropriately “urge caution in the use of treatment-induced pathologic necrosis rate as an endpoint to judge the effectiveness of therapies in clinical trials”.

Besides the percentage of viable tumor cells and necrosis, other pathologic characteristics such as fibrosis and hyalinization observed in the resected specimens have been associated with favorable outcomes. Schaeffer et al. [15] demonstrated that the percentage of fibrosis/hyalinization, rather than necrosis or viable cells, serves as a prognostic factor for LRFS and OS following neoadjuvant RT. This finding was confirmed by Boxberg et al. [17] in an institutional study evaluating the pathologic response of 64 patients treated with preoperative RT. Reijers et al. [16] retrospectively analyzed the pathological response of 107 patients with STS tumors and did not validate the prognostic value of fibrosis/hyalinization. In our study, fibrosis/hyalinization was assessed, but we failed to confirm its prognostic significance in both univariate and multivariate analyses.

Finally, it has been hypothesized that a combination of viable tumor cells, fibrosis, and other treatment-related changes may have greater prognostic value than any single characteristic alone. In this regard, Stacchiotti et al. [20], proposed that viable tumor cells should be evaluated when at least 10% of treatment-related changes, including fibrosis but excluding necrosis, are present. They categorized patients as “very good responders” when ≤10% viable tumor cells are found in the specimen, “good responders” when >10% and ≤50% viable tumor cells are found in the specimen, and “non-responders” for those not meeting these criteria. However, when we applied this classification system to our data, it did not correlate with oncological outcomes.

There are several limitations to our study that should be acknowledged. The retrospective design may have introduced biases in data collection and interpretation. Although our sample size was adequate for exploratory analysis, it is too small and heterogeneous to draw definitive conclusions, particularly when stratifying by specific histologic subtypes. However, given the rarity of STS tumors, this sample size remains relatively robust for a single-institution study. Our analysis was based on hazard ratios obtained from corresponding survival curves that extended over 5 years for a smaller group of patients. However, the median follow-up of 26 months in our cohort does not qualify as long-term follow-up. This study focused exclusively on patients who underwent a specific treatment regimen, limiting the generalizability of our findings to those who received different therapies, such as chemotherapy or combined chemoradiotherapy. Lastly, the low incidence of certain outcomes in our cohort, while suggesting effective treatment, also makes it difficult to identify prognostic factors related to these outcomes.

Despite the limitations, this study contributes valuable data to the ongoing discussion regarding the prognostic value of pathologic responses following neoadjuvant radiotherapy for soft tissue sarcomas. The findings underscore the complexity of using pathologic response as a reliable predictor of oncologic outcomes.

## 5. Conclusions

In this study, we evaluated the pathologic response of STS tumors after neoadjuvant RT using the EORTC–STBSG score and investigated its correlation with the oncological outcome. In the studied population the local recurrence risk was associated with both resection margin and tumor grade, with R1 resection margins and higher tumor grades each increasing the risk of local recurrence. High grade tumors, and tumors larger than 10 cm were associated with an increased risk of distant metastasis. Tumor size and MFS histologic subtype were unfavorable prognostic factors for OS. Resection specimens with ≤5% viable cells were linked to improved LRFS and OS. Classifying pathologic response of the analyzed specimens using the EORTC–STBSG score failed to show an association with clinical outcomes. While the EORTC–STBSG score provides a valuable framework for assessing pathologic response in soft tissue sarcomas following neoadjuvant treatments, its role as a prognostic tool remains uncertain. Further large prospective studies, ideally histology-tailored, are mandatory to investigate and validate the potential of pathologic parameters as reliable predictors of clinical outcome.

## Figures and Tables

**Table 1:** Demographic and clinicopathological characteristics of the studied patient cohort.

Characteristic	*n* = 44 ^1^
Age at diagnosis (years)	68 (56–74)
Sex	
Female	20 (45%)
Male	24 (55%)
Tumor location	
Upper extremity	5 (11%)
Lower extremity	38 (86%)
Trunk	1 (2%)
Tumor size (cm)	
≤10	23 (52%)
>10	21 (48%)
Histologic subtype ^2^	
Undifferentiated Pleomorphic Sarcoma (UPS)	23 (52%)
Myxoid Liposarcoma (MLS)	7 (16%)
Myxofibrosarcoma (MFS)	6 (14%)
Other	8 (18%)
Tumor grade	
Low	8 (18%)
High	36 (82%)
Radiotherapy technique ^3^	
3DCRT	27 (61%)
VMAT	17 (39%)
Resection margin	
R0	33 (75%)
R1	11 (25%)

^1^ Median (IQR); *n* (%). ^2^ Other histologic subtypes included: liposarcoma, dedifferentiated liposarcoma and malignant peripheral nerve sheath tumor. ^3^ 3DCRT: 3D Conformal Radiation Therapy, VMAT: Volumetric Modulated Arc Therapy.

**Table 2:** Pathologic characteristics after neoadjuvant treatment and response assessment using the EORTC-STBSG score stratified by STS histological subtype.

Parameter	*n*	% Viable ^1^	% Necrosis ^1^	% Fibrosis/ Hyalinization ^1^	EORTC–STBSG Score ^2^				
					A	B	C	D	E
All patients	44	20 (6–51)	11 (2–40)	40 (22–84)	3	2	8	18	13
Histology									
UPS	23	20 (10–45)	32 (9–49)	35 (21–49)	0	2	3	12	6
MLS	7	10 (7–12)	5 (3–6)	85 (81–87)	1	0	2	4	0
MFS	6	64 (43–80)	1 (0–7)	21 (12–36)	0	0	1	1	4
Other	8	19 (4–68)	2 (0–29)	29 (21–94)	2	0	2	1	3
*p*-value ^3^		0.069	0.041	0.044	-	-	-	-	-

^1^ Median (IQR). ^2^ EORTC–STBSG response score (*n*): A. no viable cells, B. Single viable tumor cells or small clusters (<1%), C. ≥1%–<10% viable tumor cells, D. ≥10%–<50% viable tumor cells, E. ≥50% viable tumor cells. ^3^ Kruskal-Wallis rank sum test.

**Table 3:** Multivariate analysis of tumor characteristics on the oncological outcome.

Independent Variable	LRFS		DMFS		OS	
	HR (CI95%)	*p*	HR (CI95%)	*p*	HR (CI95%)	*p*
Age (y)	1.11 (1.02–1.20)	0.012	NS		1.09 (1.00–1.19)	0.045
Size > 10 cm ^1^	NS		4.67 (1.47–14.81)	0.009	17.58 (1.89–163.08)	0.012
High Grade ^2^	17.96 (0.74–43.52)	0.076	16.81 (0.67–41.13)	0.085	NS	
MFS histology ^3^	NS		NS		40.78 (0.52–319.77)	0.096
Necrosis	NS		NS		1.09 (0.99–1.21)	0.088

^1^ Tumor size ≤ 10 cm used as reference, ^2^ Low grade tumors used as reference, ^3^ UPS histologic subtype used as reference.

**Table 4:** Pathologic findings after neoadjuvant treatment for three main STS histologies.

Histology	Study	%Viable	%Necrosis	%Fibrosis/Hyalinization
UPS	This study	20	32	35
	Schaefer et al., 2017 [15]	72.5	15	5
	Cates 2019 [26]	23.2	38.5	38
	Allignet et al., 2021 [25]	10	20	50
	Boxeberg et al., 2022 [17]	20	10	30
	Reijers et al., 2023 [16]	20	40	25
MFS	This study	64	1	21
	Schaefer et al., 2017 [15]	30	0	10
	Allignet et al., 2021 [25]	60	10	20
	Boxeberg et al., 2022 [17]	40	5	20
	Reijers et al., 2023 [16]	75	0	10
MLS	This study	10	5	85
	Schaefer et al., 2017 [15]	17.5	0	35
	Allignet et al., 2021 [25]	20	0	80
	Boxeberg et al., 2022 [17]	15	0	35
	Reijers et al., 2023 [16]	10	0	58
	Lam et al., 2023 [30]	7.5	5	67.5

## Data Availability

Study data are available upon reasonable request to the corresponding author.
